# The role of immune correlates of protection on the pathway to licensure, policy decision and use of group B *Streptococcus* vaccines for maternal immunization: considerations from World Health Organization consultations

**DOI:** 10.1016/j.vaccine.2019.04.039

**Published:** 2019-05-27

**Authors:** Johan Vekemans, Jonathan Crofts, Carol J. Baker, David Goldblatt, Paul T. Heath, Shabir A. Madhi, Kirsty Le Doare, Nick Andrews, Andrew J Pollard, Samir K. Saha, Stephanie J. Schrag, Peter G. Smith, David C. Kaslow

**Affiliations:** a Initiative for Vaccine Research, World Health Organization, 20 Av Appia, 1202 Geneva, Switzerland; b Joint Committee on Vaccination and Immunisation, Public Health England, Wellington House, 133-155 Waterloo Road, London SE1 8UG, UK; c Division of Pediatric Infectious Diseases, University of Texas McGovern Medical School., 6431 Fannin Street, MSB 3.126, Houston, TX 77030, USA; d Institute of Child Health, University College London, 30 Guilford St, London WC1N 1EH, UK; e Institute of Infection and Immunity, St George’s University of London, Cranmer Terrace, London SW17 0RE, UK; f Medical Research Council: Respiratory and Meningeal Pathogens Research Unit, Faculty of Health Sciences, University of the Witwatersrand, York Road, Parktown, 2193 Johannesburg, South Africa; g Statistics Unit, Public Health England, 61 Colindale Av., London NW9 5EQ, UK; h Oxford Vaccine Group, Department of Paediatrics, University of Oxford, and the NIHR Oxford Biomedical Research Centre, Oxford OX3 9DU, UK; i The Child Health Research Foundation at the Bangladesh Institute of Child Health, Sher-E-Banglanagar, Dhaka Shishu Hospital, Dhaka 1207, Bangladesh; j Respiratory Diseases Branch, Division of Bacterial Diseases at the Centers for Disease Control and Prevention, 1600 Clifton Road, Atlanta 30333, USA; k Tropical Epidemiology Group, London School of Hygiene & Tropical Medicine, London WC1E 7HT, UK; l PATH, 2201 Westlake Avenue, Suite 200, Seattle, WA 98121, USA

**Keywords:** Group B Streptococcus, Vaccines, Correlates of protection, Maternal immunization, Neonatal sepsis

## Abstract

The development of a group B *Streptococcus* (GBS) vaccine for maternal immunization constitutes a global public health priority, to prevent GBS-associated early life invasive disease, stillbirth, premature birth, maternal sepsis, adverse neurodevelopmental consequences, and to reduce perinatal antibiotic use. Sample size requirements for the conduct of a randomized placebo-controlled trial to assess vaccine efficacy against the most relevant clinical endpoints, under conditions of appropriate ethical standards of care, constitute a significant obstacle on the pathway to vaccine availability. Alternatively, indirect evidence of protection based on immunologic data from vaccine and sero-epidemiological studies, complemented by data from opsonophagocytic *in vitro* assays and animal models, could be considered as pivotal data for licensure, with subsequent confirmation of effectiveness against disease outcomes in post-licensure evaluations. Based on discussions initiated by the World Health Organization we present key considerations about the potential role of correlates of protection towards an accelerated pathway for GBS vaccine licensure and wide scale use. Priority activities to support progress to regulatory and policy decision are outlined.

## Highlights

• There is a major public health need for GBS vaccines for maternal immunization.
• Important obstacles lie in the way of a pivotal clinical efficacy trial for licensure.
• A vaccine development pathway based on an immune correlate of protection is envisaged.
• Key considerations and priority activities for success are presented, based on WHO consultations.

## 1 Introduction

### 1.1 Burden

Group B Streptococcus (GBS) is a leading cause of maternal, foetal, neonatal and young infant invasive bacterial disease. According to recent global disease burden estimates, GBS causes 319,000 cases of early life invasive bacterial disease and 90,000 infant deaths annually. While data gaps hinder precise estimates for other disease syndromes, a conservative assessment suggests that yearly, 57,000 stillbirths are caused by GBS and 33,000 pregnant or puerperal women have GBS sepsis. There is also a significant, unquantified burden of GBS-related prematurity, neurodevelopmental impairment and maternal deaths. The African continent accounts for an estimated 54% of cases of GBS invasive disease and 65% of all foetal/infant GBS deaths [1].

The reservoir for GBS in humans is the gastrointestinal tract, and maternal recto-vaginal colonization can lead to ascending foetal infection, stillbirth and early onset neonatal sepsis. Maternal colonization in pregnancy (ranging from about 10% to 40%) has been found in women in all geographical settings evaluated [2]. Although incidence is highest during the first days of life, the risk of GBS disease in young infants extends to 3 months of life. Intrapartum antibiotic prophylaxis (IAP), guided by microbiological or clinical screening strategies, has been shown to reduce the risk of early-onset GBS disease (EOD, days 0–6 after birth) in some high-income settings, but has shown no impact on late-onset GBS disease (LOD, days 7–89) and does not prevent GBS-associated stillbirths and prematurity. There are considerable challenges to implementing effective IAP-based prevention strategies, especially in resource-limited settings, and there are concerns about unintended consequences of neonatal antibiotic exposure, including its impact on the gut microbiome and in contributing to the generation of antibiotic resistance [3].

### 1.2 Vaccine landscape

A summary of the GBS vaccine development landscape is presented in Panel 1. The development of GBS vaccines suitable for immunization during pregnancy has been identified as a priority by the World Health Organization (WHO), and key consensus documents presenting preferences for GBS vaccine product characteristics and a research and development technology roadmap have been developed [10]. Consensus-building about a preferred clinical development pathway and defining the pivotal data package requirement for licensure, and policy for vaccine use, were highlighted as priority activities.

**Panel 1** **Vaccine candidate landscape.**

Two large pharmaceutical companies, GlaxoSmithKline (GSK) and Pfizer, are building on their experience in polysaccharide-protein conjugation technology to develop GBS vaccines. Ten GBS capsular envelope polysaccharide (CPS)-based serotypes have been described, however, the majority of invasive disease in young infants is caused by a few of these serotypes (predominantly Ia, Ib, II, III, IV, V) [4]. Both companies have developed candidate vaccines targeting the major serotypes associated with neonatal disease, using the CRM197 diphtheria protein for conjugation. Novartis, which has been acquired by GSK, developed a trivalent conjugate vaccine (Ia, Ib, III), which showed favourable safety and immunogenicity in phase I and II studies in non-pregnant and pregnant women [5]. Opsonophagocytic bacterial killing activity in umbilical cord sera, correlating with serotype-specific IgG antibody concentrations, and functional activity in a mouse bacterial challenge model, were demonstrated [6]. Undetectable serotype-specific capsular antibodies at baseline and maternal HIV infection were, however, associated with reduced immunogenicity [7]. Vaccination in pregnancy was shown not to inhibit offspring responses to recommended vaccines comprised of CRM197-based conjugates [8]. GSK is back to preclinical, reformulating the candidate vaccine, while Pfizer is developing a hexavalent formulation (Ia, Ib, II, III, IV, V). A Phase I/2 clinical study in the US has been completed in healthy adults (NCT03170609) and a trial in pregnant women in South Africa, co-funded by the Bill and Melinda Gates Foundation (BMGF), isbeing initiated (NCT03765073).

A BMGF-funded partnership has also been announced between PATH and The Biovac Institute, a South-African government-private partnership, aimed at developing in-country manufacturing capacity. This vaccine development programme will aim for the development of a low-cost conjugate GBS vaccine against serotypes causing >90% of invasive GBS disease in Africa.

Minervax, in contrast, is developing a protein-based GBS vaccine, targeting the N-terminal domains of the Alp-protein family. The induction of functional activity and protection from protein-based GBS vaccines have been investigated in preclinical infection models, and further sero-epidemiological studies are required to confirm whether immunity targeting these proteins derived from natural exposure is associated with protection against early life invasive disease [9]. Minervax has completed a phase 1 trial in non-pregnant women 18–40 years of age (NCT02459262) but results have not been published to date. Another Phase 1 trial is ongoing (NCT03807245).

### 1.3 Randomized controlled vaccine clinical efficacy trial: a sample size challenge

The conduct of double-blind placebo-controlled randomized trials to assess vaccine efficacy against clinically relevant, pre-defined, endpoints constitutes the gold-standard approach to generate evidence for vaccine licensure and policy decisions. However, a pivotal GBS vaccine efficacy trial would require a very large sample size, as shown in Table 1. Baseline incidences of invasive GBS disease of 1–3 events per 1000 live births have been reported in some low-resources settings [3], but baseline incidence in a trial will depend on standards of care and subject selection criteria. In a trial, ethical considerations would require implementation of national and non GBS-specific WHO recommendations for infection prevention and strengthening of access to pre- and peri-natal care, ensuring appropriate medical oversight of delivery and prescription of intrapartum antibiotics in the presence of infectious risk factors, which would reduce baseline attack rates. It seems unlikely that the baseline incidence of eligible endpoint cases would be much higher than 0.5–1 per 1000 live births, for a trial conducted in the context of fully implemented local healthcare guidelines and optimal access to care. Candidate vaccines against other pathogens have been tested in very large pivotal trials before, but specificities related to GBS, including vaccination of women during pregnancy, the extremely rapid progression of GBS sepsis before and soon after birth, follow up requirements for women in late pregnancy, for babies in the first days and weeks of life, the need to investigate stillbirth and fatal cases, the needs for adequate safety oversight and efficacy monitoring requiring invasive sampling (blood and CSF) and bacteriologic analyses would make such a GBS vaccine trial with clinical endpoints lengthy and very costly. This traditional clinical development pathway would constitute a potential major obstacle to progress towards availability of a much-needed vaccine.

**Table 1:** Total number of pregnant women required in a placebo-controlled trial to demonstrate the efficacy of a GBS vaccine candidate against a defined disease endpoint.

Vaccine efficacy trial is designed to detect (with a lower limit of 95% confidence interval of 25%)	Expected disease rate in placebo recipients (cases per 1000 livebirths)			
	2.0	1.0	0.5	0.1
80%	30,000	62,000	122,000	620,000
60%	90,000	180,000	360,000	1,804,000

Note: Method according to Farrington, 1990 [11].

Assumptions: 80% power, P < 0.05 for significance, 1:1 vaccine:placebo allocation, 15% loss to follow-up, 90% cases eligibility for inclusion as per primary case definition, 95% matching between vaccine and circulating types.

### 1.4 Alternative pathway via correlates of protection

If a pre-licensure pivotal randomized controlled clinical efficacy trial is not possible, on grounds of cost or feasibility, alternative strategies to support licensure need to be considered. The aim of this report is to highlight the potential role that immune correlates of protection may have for an accelerated GBS vaccine development pathway to licensure and policy decision. Related, priority activities to are outlined in Table 2. The considerations presented here were informed by a consensus-generating consultation process that started with a meeting of experts and specific stakeholders, organized by WHO in London on Dec 16th, 2017, and further iterative exchanges with experts and stakeholders in the field. These considerations aim to inform vaccine development strategies, complementing recent discussions in other fora, including those organized by the US Food and Drug Administration and the UK Joint Committee on Vaccination and Immunization [12], [13].

**Table 2:** The role of immune correlates of protection: key activities on an accelerated pathway to GBS vaccine licensure, policy decision and global use.

1. Networks of investigators able to deliver high quality data according to Good Clinical Practices are developed, including research centres from diverse geographical areas. Baseline data are collected, supporting detailed epidemiological characterization and preparing for high standards in data collection and study procedures. Clinical management algorithms and standards of care are defined.
2. High quality standardized functional immune assays, measuring bactericidal activity in serum, are developed; antigen binding assays that closely correlate with functional activity are developed.
3. Study protocols aimed at characterizing the relationship between antibody concentrations and disease risk in a non-vaccinated, naturally exposed population, are developed.
4. Detailed analysis plans based on threshold or continuous model analysis are pre-established, defining the primary analysis and secondary exploratory analyses. Sample timepoints of analysis are predefined. Specific and sensitive clinically relevant endpoints are pre-defined.
5. Large-scale sero-epidemiological studies are conducted across diverse geographical settings, designed specifically to support derivation of correlates of protection.
6. Estimates of effects on the protective function of antibodies are produced, according to predefined analytical plans. When possible, serotype/strain specific estimates of effects are produced. Factors affecting the association are characterized. Aggregate estimates across serotypes/strains are generated.
7. Maternal vaccination trials are conducted according to predefined protocols and analytical plans.
8. Favourable safety is established, to international quality standards.
9. Success criteria are pre-defined: vaccination induces antibody levels above protective thresholds in a high, predefined proportion of recipients (or alternative robust statistical estimates based on continuous models). Aggregate estimates of effects are produced, serotype/strain specificity is investigated. Antibody persistence is demonstrated, beyond the period-at-risk. Pre-defined success criteria are passed.
10. Factors affecting immunogenicity and antibody transfer are characterized.
11. Vaccine induced antibodies are shown to be functionally active in bactericidal assays. Serotype/strain specificity is investigated.
12. Various animal models of protection suggest vaccination protects against experimental infection. Serotype/strain specificity is investigated.
13. Initial licensure is obtained based on indirect evidence and agreement on necessary post-licensure Phase 4 effectiveness studies
14. Plans for confirmatory evaluation of public health impact based on consensus study design are developed early and financed.
15. Post-licensure pilot implementation studies are conducted without delays, leading to policy decision for wide-scale use, country processes start, and procurement is ensured by public health agencies, informed by implementation science and analyses of full public vaccine value.

General WHO considerations on methods leading to definitions of correlates of vaccine-induced protection have been presented elsewhere [14]. Accordingly, in terms of nomenclature, we here refer to a substitute endpoint as a non-clinical pivotal endpoint which may be used to support licensing decisions, and to immune correlates of protection as immune markers shown to correlate statistically with a reduced risk of disease. Such a correlate may, or may not, be a causal mediator of protection. The term ‘surrogate endpoint’, specifically implies that the measured marker is a causal, mechanistic mediator of protection.

### 1.5 Past experience on the role of correlates of protection on the pathway to vaccine availability

Correlates of protection have played an important role in supporting the licensure of several conjugated vaccines targeting encapsulated bacteria [15], [16], as reviewed in Panel 2. The type of studies that have contributed to establishing evidence of correlates of protection have included sero-epidemiological studies, showing associations between antibody levels and subsequent incidence of disease, passive immunization experiments with serum transfer in animal models or humans showing protection; animal models of vaccine-induced protection; *in vitro* bacterial killing assays; and ultimately human efficacy, effectiveness studies.

When putting into perspective past experience with other vaccines and considerations on substitute immunological endpoints for GBS, important similarities and differences can be highlighted. Often, the mechanism of immunity derived from natural exposure and vaccine-induced immunity is similar, and based on functional antibodies, but protective thresholds can differ according to antibody qualitative characteristics. Thresholds may also vary by pathogen strain/serotype and clinical syndrome. In all the non-GBS examples discussed in Panel 2, some efficacy data were part of the evidence package used for licensure, although not necessarily specific to all antigens included in the vaccine. Sometimes the efficacy evidence was derived by analogy to a related vaccine rather than generated from the candidate vaccine itself. Relative to other diseases with extended time-at-risk, the time window of susceptibility to invasive GBS disease in babies is narrow, which should allow a more specific evaluation of the relationship between antibody threshold and protection over time. Another important difference relates to the fact that adaptive immunity in babies is not expected to play a role in early life protection against GBS, as protection would be passively derived only from acquisition of maternal antibodies.

**Panel 2** **Learning from history: The role of correlates of protection on the development pathway of selected available vaccines**

As early as 1933, epidemiological studies of *Haemophilus influenzae* type b (Hib) infections showed that serum bactericidal activity (SBA) increased with age [17]. SBA was later found to be correlated with the level of IgG antibody against the organism’s CPS, allowing serum ELISA concentrations to be considered as a useful marker associated with protection [18]. Subsequent efficacy studies of a Hib polysaccharide vaccine candidate led to the demonstration of an association between vaccine-induced antibodies and protection from natural Hib exposure, and the definition of protective thresholds [19]. CPS conjugation with a carrier protein increased immunogenicity and functional activity *in vitro*, and a lower antibody threshold of protection than for the polysaccharide vaccine. Post-immunization with a protein conjugate Hib CPS vaccine, a peak antibody concentration of 1 μg/mL is considered as a marker of long-term protection. Prevalent concentrations above 0.15 μg/mL are considered as protective against Hib bacteraemia and are accepted as such by regulators for licensure of new vaccines or for bridging studies [15]. Higher levels of antibodies have been associated with protection against nasopharyngeal carriage [20].

Epidemiological studies in the 1960′s similarly showed an association between age and anti-capsular groups A, B and C meningococcus serum complement-dependent bactericidal assay (SBA), and between the latter and the risk of meningitis [21]. Vaccine efficacy trials leading to licensure and introduction of polysaccharide meningococcal A and A/C vaccines enabled correlate of protection to be defined. Quadrivalent formulations, including serogroups W and Y in addition to A and C, were marketed internationally with no direct evidence of efficacy for the W and Y serotypes. Serological criteria alone without direct evidence of efficacy supported the introduction of the meningococcal C conjugate vaccine in the United Kingdom in 1999, and it was only when early post-licensure evaluation produced efficacy estimates that the licensure criteria were clinically validated [22]. A serogroup A meningococcal polysaccharide-tetanus toxoid conjugate vaccine was licensed in India in 2009, and pre-qualified by WHO in 2010, on safety and immunogenicity (SBA assays) data only. The vaccine was then deployed in the sub-Saharan Africa meningitis belt and shown to be protective against meningococcal meningitis [23].

More recently, a non-capsular, outer membrane protein (OMP)-based meningococcal group B vaccine was licensed, based on indirect evidence of efficacy. Vaccine efficacy had been demonstrated in the 1990s for two OMP-based vaccines derived from outer membrane vesicles (OMV), and the association with hSBA responses (h indicative of human complement use) had been shown [24]. However, the highly variable nature of the immunodominant vesicular PorA protein limited strain coverage, and genomic advances in the 2000′s supported the development of new protein vaccine candidates. In 2013, MenB-4C was licensed in Europe, based on demonstrated SBA against a panel of strains matched to the vaccine antigens in the absence of direct efficacy data. In 2015, MenB-4C was introduced in the routine infant vaccination schedule in the UK. In 2015, the MenB-FHbp and MenB-4C protein vaccines were licensed by the US FDA, based on regulations for accelerated approval, which supports approval of vaccines for serious or life-threatening diseases based on safety and indirect evidence reasonably likely to predict clinical benefit, in this case hSBA responses. As a requirement, post-marketing confirmatory effectiveness studies were to be conducted, including investigating herd protection and evaluating the impact of vaccination on strain variation and carriage [25]. Post-licensure evidence of effectiveness soon emerged [26].

Randomized controlled trials initially established the protective efficacy of the 7-valent pneumococcal conjugate vaccine (PCV), which allowed the derivation of serotype-specific serum antibody protective thresholds for some serotypes, but not for the less common ones. Although there was no evidence demonstrating that protective thresholds were the same for different serotypes, it was considered acceptable to estimate an aggregate threshold through data pooling, serving as target for all serotypes [27]. The aggregate threshold was used for licensure of subsequent PCV vaccines, using head-to-head immunogenicity studies for the same serotypes, and as a stand-alone reference for the additional serotypes when new capsular types were included. Post-licensure, serotype-specific protective thresholds documented from vaccine effectiveness studies showed some variation relative to the initially inferred aggregate correlate of protection [28].

Correlates of protection were initially established in efficacy studies of PCV against all invasive pneumococcal disease (including bacteraemia and meningitis), but true threshold might differ according to disease entities. Protective levels associated with protection against acute otitis media may be higher [29]. Results from a study of maternal-foetal transfer of antibodies acquired following natural exposure suggested that even higher antibody levels are needed to prevent pneumococcal carriage in infants [30].

## 2 Establishing a valid correlate of protection for the licensure of GBS vaccines: available evidence, ongoing studies

### 2.1 Current evidence base

The first suggestion that it may be possible to establish a serological correlate of protection for GBS was presented four decades ago [31]. A number of studies have since reported a reduced risk of invasive disease in neonates and young infants associated with higher maternal antibodies acquired following natural exposure [32], [33], [34], [35], [36], [37], as presented in a recent review [38]. In most studies, ligand-binding or Luminex® assays were used; in some, opsonophagocytic *in vitro* killing assays were used. While antibody-mediated risk reduction estimates have been reported from different studies for the most frequent serotypes (Ia, III, V), rigorous estimates of protective thresholds have not been established. There were important differences in the design of the various studies, which were conducted across diverse populations and epidemiological settings, with different standards of care, studying different target antigens, using different reference sera and other control reagents, assay methodologies, analytical methods, and length of follow-up. An inverse association between levels of antibodies and neonatal risk of invasive disease has also been shown for some protein candidate antigens, but not others [37], [39], [40]. In addition, other studies aiming to characterize correlates of protection are currently underway or being planned.

### 2.2 Ongoing or planned sero-epidemiology studies

In South Africa, a prospective, observational cohort of 35,000 mother-newborn pairs was enrolled between 2015 and 2017. Enrolment was re-initiated in 2019, to include a further 15,000 pairs. Infants were followed-up by hospital-based surveillance for invasive GBS disease. In parallel, infants with invasive disease that were not enrolled into the birth cohort were, and will further be, identified across multiple hospitals. The study aims to be powered for a case-control evaluation of sero-correlates of protection for serotype Ia and III, the most common serotypes causing invasive disease in South Africa. Cases will be matched to controls for maternal colonization status with a homotypic serotype during labour, maternal age, birth weight, newborn gender and maternal HIV status (Shabir Madhi, personal communication).

A case-control study with similar objectives is also planned in the United States by the Centers for Disease Control and Prevention (CDC). The association between GBS serotype-specific capsular IgG antibody concentrations at birth and odds of invasive GBS disease will be estimated, for serotypes Ia and III separately. Cases will be identified in 2019–2022 from CDC’s Active Bacterial Core surveillance program. Controls will be babies of GBS-colonized mothers identified at participating clinical centres performing routine antenatal screening for GBS colonization. Remainders of the newborn screening dried blood spot (DBS) samples will be used for antibody measurements. The study aims to enrol approximately 500 cases and 3000 controls (Stephanie Schrag, personal communication).

Pilot studies are underway both in the UK and Uganda to assess the feasibility of prospectively enrolling a high number of mother-infant pairs and collect maternal and cord sera (150.000 in the UK, 35.000 in Uganda), using similar methodologies. All pairs are planned to be followed up to 90 days after birth. In case of disease, another maternal and infant sample will be taken and antibody titres compared to those at birth. Controls will be selected from both GBS-colonized and GBS non-colonized women matched on gestation and gender, aiming for at least 3 controls for every case. A further retrospective study from 2014, based on stored DBS samples, is also underway, using serum samples from 150 cases and three times as many controls (Kirsty Le Doare, personal communication).

### 2.3 Animal models

In addition to sero-epidemiological studies, studies in relevant experimental animal models can contribute to establishing evidence of a protective role of antibodies induced by vaccination. Such models have provided useful mechanistic insights into the pathogenesis and the impact of antibody-mediated responses on GBS disease. Passive transfer of antibodies targeting the polysaccharide envelope has provided serotype-specific protection from experimental GBS challenge [41]. Passive transfer of antibodies acquired following experimental infection has also been shown to be partially protective in rhesus monkeys [42]. Animal models have also been used to study the protective effect of maternal immunisation on the offspring and have demonstrated placental antibody transfer and favourable outcomes, for both capsular envelope polysaccharide and protein-based candidate vaccines [37], [43], [44]. Maternal vaccination in non-human primate models has been shown to induce high maternal antibodies and results in transfer of functional antibodies to offspring [45].

## 3 Defining the way forward

Upon demonstration that a traditional clinical development pathway is unfeasible at a reasonable cost, alternative approaches should be considered. Whatever the approach, strong evidence establishing the validity of substitute endpoint(s) as reliable predictors of clinical benefit will be required to support regulatory and policy decisions. Important consensus recommendations about the use of an immunological substitute endpoint are highlighted here, and an analysis framework for critical appraisal of evidence is presented in Panel 3.

Panel 3 **Key considerations in defining and validating a substitute endpoint**

The most important determining factor for the accelerated approval of a GBS vaccine based on a non-clinical endpoint will be how strongly the available package of evidence is graded as reasonably likely to predict clinical benefit.

The Prentice criteria have been proposed to assess the validity of proposed substitute endpoints [46]. The first requirement is that vaccination affects outcome. The proposed accelerated approval pathway assumes that this can only be inferred indirectly and cannot be demonstrated pre-licensure and should be demonstrated after initial vaccine introduction. The second criterion is that vaccination affects surrogates (in this case antibodies), which can be demonstrated in vaccine immunogenicity studies. The third criterion is that surrogates affect outcome. This would need to be established in sero-epidemiological studies. The fourth criterion is that the conditional disease risk (the association between the surrogate outcome and the disease risk) is the same in vaccinated and unvaccinated individuals. The full mediation condition relates to the observation that the substitute variable is a full mediator of protection. In this situation, whereby we want to extrapolate results from immunity studies in conditions of natural exposure with immunity resulting from vaccination, this assumes that the protective mechanism is the same in both conditions (non-vaccinated previously exposed and vaccinated), or is sufficiently close. This relies on the assumption that functionality of antibodies induced by vaccination and natural exposure are the same (or closely associated). Assessing this requires an understanding of the underlying biological mechanism, and a causality assessment of the link between the substitute endpoint and the clinical endpoint of ultimate interest.

The Bradford-Hill criteria have been proposed to support causality assessments: 1. Temporal sequence of association (exposure triggers appearance of antibodies, the role of which precede the clinical outcome); 2. Strength of association (to be determined in sero-epidemiological studies); 3. Consistency of association (shown through repeat studies in different settings); 4. Biological gradient (as to be determined in sero-epidemiological studies); 5. Specificity (a critical criteria, as other trans-placentally transferred protective immune effectors that are not induced by vaccination could lead to an over-estimate of the potential impact of vaccines); 6. Plausibility (supported by other maternal immunization platforms and by other vaccines targeting encapsulated bacteria); 7. Coherence (the causality assumption does not appear to conflict with current knowledge); 8. Experimental reversibility (upon waning of immunity acquired post exposure); 9. Analogy (supported by other maternal immunization platforms and by other vaccines targeting encapsulated bacteria).

Based on this analysis framework, a functional antibody assay would seem preferable as a scientifically justifiable substitute endpoint to just a quantitative, non-functional antigen-binding assay. Alternatively, strong evidence of the correspondence between functional and non-functional quantitative assays should be demonstrated. Animal models of vaccine-induced protection would also add to the evidence that the measured antibodies are clinically relevant.

### 3.1 Safety

The safety evaluation of a candidate vaccine needs to be at least as rigorous as that required for vaccines licensed by traditional clinical development pathways. When considering risk-benefit, the uncertainty about the benefit derived using a substitute endpoint for licensure might justify setting a higher safety profile expectation. Considerations about pregnancy immunization safety characterization are presented elsewhere [47].

### 3.2 Endpoint case definitions

Standard case definitions and ascertainment methodologies are needed to produce clinically relevant estimates of effect for both the evaluation of vaccine efficacy against a clinical endpoint, and for the evaluation of association between antibody levels and clinical outcomes in a sero-epidemiologic study. WHO preferred case definitions and ascertainment methodologies are described elsewhere [48]. Briefly, a systematic case detection system should be implemented to support identification of confirmed GBS cases. The use of a composite endpoint, including different clinically relevant disease entities (e.g., GBS-confirmed stillbirth, EOD, LOD, and fatal invasive disease) may need to be considered to reduce study size requirements. Case detection systems need to be applied from late pregnancy or at the time of birth, as most cases of EOD occur on the day of birth. The clinical management algorithm and clinical criteria used to trigger collection of a clinical sample for GBS culture will play a critical role in identifying cases. Criteria used to trigger sample collection should be set to maximize case capture. Highest standards of sterile technique in sample collection should be applied, to avoid sample bacterial contamination and maximize sensitivity, which is drastically reduced if an insufficient blood volume is collected.

The role of GBS maternal or neonatal colonization as a potential substitute endpoint has been discussed elsewhere and is outside the scope of this report [48]. In the context of a sero-epidemiological study to define serological correlates of protection, detection of maternal recto-vaginal colonization and neonatal colonization and bacterial typing is encouraged, as a secondary stratified analysis considering colonization status may contribute to data interpretation.

### 3.3 Assay quality systems

High quality standards in assay quantification of antibody level and function should apply. Characterization of the performance of the assay need to be adapted to the vaccine development status, noting that an immunological substitute endpoint should ultimately be based on fully analytically validated assays. International agreement on standards and methodologies will provide the necessary support for comparison and bridging studies. A collaborative initiative towards the development of validated standardized immunoassays is ongoing [49].

### 3.4 Sampling timepoints

The choice of sampling timepoints in both mothers and offspring will be a key determinant of the evaluation of the association between antibody levels and protection. Samples from both the mother and the offspring should be analysed. Several options could be considered to define the primary time-point used to assess protection thresholds. The analysis of a maternal post-immunization serum sample may constitute a pragmatic approach to capture an overall target level that would be associated with a reduction of the overall risk of adverse outcomes, including stillbirth, maternal and infant outcomes. However, sources of heterogeneity need to be considered, as the gestational age at vaccination, prematurity and co-morbidities affect placental transfer of antibodies. Thresholds may also vary according to clinical outcomes considered. Antibody levels in cord blood provide an opportunity to define, possibly with more specificity and precision, a functional protective antibody threshold – the level of antibodies that is required to prevent invasive disease in the offspring, upon initial bacterial penetration into the blood stream. Further research is necessary to understand whether neonatal antibody levels measured may be lowered, in the context of invasive infection, by bacterial binding of antibody, and implications for data interpretation. The characterization of antibody persistence over time up to the end of the at-risk period may also contribute to assess the relationship between antibody levels and disease risk, especially for late-onset disease.

In vaccine studies, antibody levels at birth should be evaluated as a function of timing of maternal immunization during pregnancy, for vaccination program planning.

### 3.5 Statistical considerations

Important statistical considerations driving the prospective development of analysis plans need to be defined. WHO has expressed a preference for an 80% level of protection provided by GBS vaccination [10], and this should guide sample size determination for an approach using immune correlates of protection. The target effect size and a detailed analysis plan defining the primary outcome of interest and key secondary analyses should be prospectively defined. The respective advantages and limitations of various statistical analysis methodologies should be considered. Whether a threshold or continuous model-based approach is favoured should be predefined [50].

Geographical heterogeneities should be considered, as the protective threshold may differ according to various host and pathogen factors. Statistical evaluations should also be undertaken in consideration of other variables that may impact the validity of the assumptions. The number, intensity and duration of prior GBS infections, comorbidities, the microbiome, host genetics, or pathogen characteristics may impact the type of immune response generated after natural infection as compared to a response to vaccination, with potential consequences on antibody transfer. Statistical models supporting the assessment of the role of multiple variables may contribute to a better characterization of the strength of the assumptions. Exploratory evaluations can contribute to characterize the role of phenotypic and genotypic differences in the pathogen with protection, thereby producing valuable knowledge to inform vaccine development pathways and decision-making.

If the vaccine-induced antibody levels are far above the antibody protective threshold defined by studies in conditions of natural exposure, the likelihood that the vaccine is protective will be higher. A risk management plan should be considered if a significant proportion of vaccinated individuals are categorized as low responders, which may justify investigating a two-dose vaccine regimen, possibly including a pre-pregnancy priming dose.

A concern, regarding the validity of extrapolating a threshold indicative of antibody-mediated immunity resulting from natural exposure to post vaccination levels, relates to the possibility that other protective immune mediators, activated by natural exposure (ie antibodies targeting other antigens), are transferred to the foetus, but may not be induced by vaccination. In theory, this effect could be complete, or only partial, if the measured antibody is just one mediator of protection amongst others. Such a situation may lead to an overestimation of the likely vaccine efficacy based on the substitute immunological endpoint. A way to mitigate this concern would be to build a strong data package indicative of the direct protective role of the substitute immune endpoint effector (i.e., ideally establishing that the immune response is both necessary and sufficient for protection), or of the close association between the measured endpoint and the causal mediator of protection. For instance, confidence in the extrapolation of immunity from natural exposure to immunity resulting from vaccination can be increased by *in-vitro* demonstration that the measured antibody is functional, through measurement of bacterial killing assays, and by animal models of protection.

### 3.6 Rare serotypes

Even if it were possible to conduct a large randomized controlled trial testing the efficacy of a multivalent polysaccharide-protein conjugate candidate vaccine, such a trial may generate an overall vaccine efficacy estimate, and possibly serotype-specific efficacy estimates for the most frequent serotypes. It is unlikely that reliable vaccine efficacy estimates would be produced for less common serotypes. Similarly, epidemiological studies in conditions of natural exposure may generate estimates of protective serological thresholds against the most frequent serotypes, but not the less common ones. As has been done for other multivalent vaccines, an aggregate threshold may need to be derived, based on a weighted average, observations about threshold differences across common serotypes and some plausible assumptions. Later, subsequent post-licensure investigations may allow more detailed characterization.

### 3.7 Post licensure requirements

An inherent disadvantage of an approach based on a substitute endpoint relates to the imprecise characterization of the individual and public health impact of the vaccine, better estimated through direct evidence generated in a large double blind randomized controlled trial. In the absence of such evidence pre-licensure, impact estimates must rely on mathematical models prone to imperfect assumptions.

In such a context, regulators are likely to consider sero-correlate-based indirect evidence of protection only in the context of agreed post-approval studies for further evaluation of effectiveness and impact. Licensure may be conditional, subject to review after the conduct of such studies. Policy makers would also likely require studies to assess effectiveness and impact before advising in favour of prioritizing resources for large-scale implementation. Post-licensure studies offer the possibility to further assess the public health impact of the vaccine, including its preventive role against less common endpoints, such as mortality or multi-factorial syndromes like prematurity. Such studies may include cluster-randomised trials, stepped-wedge designs for vaccine introduction, case-control studies or ecological studies monitoring changes in population disease burden. They may be linked to evaluations of vaccine programme implementation feasibility, as these are increasingly seen as necessary on the pathway to vaccine availability, especially in resource-limiting settings with frail health systems, where deployment of new interventions is most challenging, and where competition for scarce resources is most prevalent [51], [52]. Early planning and upfront identification of financing mechanisms for pilot implementation and post-licensure evidence generation should be identified, to avoid unnecessary delays in access to life-saving interventions in the populations most in need.

## 4 Conclusions

There is an urgent need for new tools to prevent the morbidity and mortality associated with GBS disease, and GBS vaccine development is a leading priority for protection of early life. While randomized controlled clinical efficacy trials constitute the gold standard for evidence generation, there are significant obstacles to conducting such trials to assess efficacy against GBS invasive disease.

Alternative strategies to accelerate licensure of candidate vaccines must be explored, including efforts to define serological correlates of protection as acceptable substitute endpoints, derived from studies of immunity resulting from natural exposure. Existing evidence suggest this may be a viable approach. Vaccine development sponsors, advised by regulators and policy makers, will need to decide on their preferred vaccine development pathway for licensure and policy decisions, through a careful analysis of risks and opportunities. Multiple stakeholders should be engaged early to promote strategic alignment and agree prospectively on methodological preferences, data package requirements and success criteria, tackling priority activities outlined in Table 2.

Any evaluative approach is likely to include important assumptions and gaps. For instance, it will be hard to generate evidence about less common serotypes. Sources of geographical heterogeneities should be considered. A global strategy should be developed, and similar studies should be undertaken in high-, low- and middle-income country settings. Protective thresholds may differ according to local epidemiology and host and pathogen factors.

From an early stage in vaccine development, regulators and policy makers should work with sponsors on possible vaccine development pathways. A pathway involving regulatory decision-making based on indirect evidence of clinical impact will require post-licensure confirmation of public health benefits associated with vaccine use in large populations, possibly during a pilot implementation stage. Early planning of confirmatory evaluation according to agreed methodologies, with identified financing, will be key to reduce delays to wide-scale use, including in low resource settings.
